# Image fusion for dynamic contrast enhanced magnetic resonance imaging

**DOI:** 10.1186/1475-925X-3-35

**Published:** 2004-10-19

**Authors:** Thorsten Twellmann, Axel Saalbach, Olaf Gerstung, Martin O Leach, Tim W Nattkemper

**Affiliations:** 1 Applied Neuroinformatics Group, Faculty of Technology, University of Bielefeld, Germany; 2 Cancer Research UK Clinical MR Research Group, Section of Magnetic Resonance, Institute of Cancer Research, Royal Marsden Hospital, Sutton, Surrey, UK

## Abstract

**Background:**

Multivariate imaging techniques such as dynamic contrast-enhanced magnetic resonance imaging (DCE-MRI) have been shown to provide valuable information for medical diagnosis. Even though these techniques provide new information, integrating and evaluating the much wider range of information is a challenging task for the human observer. This task may be assisted with the use of image fusion algorithms.

**Methods:**

In this paper, image fusion based on *Kernel Principal Component Analysis *(KPCA) is proposed for the first time. It is demonstrated that a priori knowledge about the data domain can be easily incorporated into the parametrisation of the KPCA, leading to task-oriented visualisations of the multivariate data. The results of the fusion process are compared with those of the well-known and established standard linear *Principal Component Analysis *(PCA) by means of temporal sequences of 3D MRI volumes from six patients who took part in a breast cancer screening study.

**Results:**

The PCA and KPCA algorithms are able to integrate information from a sequence of MRI volumes into informative gray value or colour images. By incorporating a priori knowledge, the fusion process can be automated and optimised in order to visualise suspicious lesions with high contrast to normal tissue.

**Conclusion:**

Our machine learning based image fusion approach maps the full signal space of a temporal DCE-MRI sequence to a single meaningful visualisation with good tissue/lesion contrast and thus supports the radiologist during manual image evaluation.

## Background

**Figure 1:**
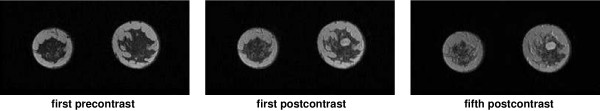
Visualisation of contrast agent concentration as gray value images of the same volume slice at different points of time (Left to right: first precontrast, first postcontrast and fifth postcontrast image). The lesion is located near the centre of the right breast.

**Figure 2:**
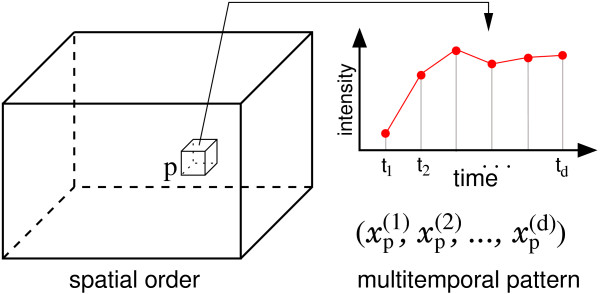
Alternative view on a temporal sequence of *d *3D MRI volumes: Each spatial coordinate **p **in a 3D volume can be associated with a *d*-dimensional temporal kinetic vector **x**_**p **_consisting of measurements of the local intensity at *d *points of time.

**Figure 3:**
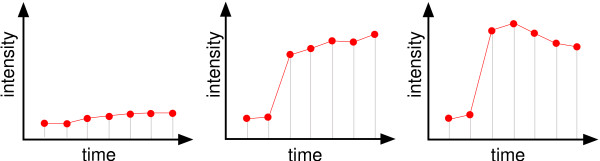
Illustration of temporal kinetic patterns of contrast uptake for normal, benign and malignant tissue (left to right) measured during DCE-MRI with two precontrast and five postcontrast recordings. Especially the strong signal uptake between the two precontrast measurements and the first postcontrast measurement indicates suspicious tissue.

In recent years, multivariate imaging techniques have become an important source of information to aid diagnosis in many medical fields. One example is the *dynamic contrast-enhanced magnetic resonance imaging *(DCE-MRI) technique [1,2]. After the administration of a gadolinium-based contrast agent, a sequence of *d *3D MRI volumes is recorded from a certain part of the body (see Fig. 1). Thus, each spatial coordinate **p **= (*x*, *y*, *z*) in the volume can be associated with a temporal kinetic pattern vector  which is regarded as a point in a signal space  (see Fig. 2). The examination of these temporal kinetic patterns at different spatial coordinates in the volume allows the observer to infer information about local tissue types and states (see Fig. 3) [3].

Today, much effort is spent on enhancing the capabilities of the imaging techniques e.g. increasing the spatial and temporal resolution. In contrast to these improvements in image acquisition, much less effort has been spent on effective visualisation methods. Even though several approaches for detection and classification of suspicious lesions in DCE-MRI data of the breast have been proposed (e.g. [4-8]), it is still common practice for the huge amount of data to be analysed manually using simple operations such as *subtraction images *of two volumes.

Obviously, these images can only comprise a small fraction of the information which is commonly spread over all volumes of the sequences. As a consequence, analysing multivariate images in radiology remains a time consuming and challenging task which potentially can be alleviated by the application of *image fusion *techniques.

### Image fusion

Image fusion methods have been an area of research for several decades. According to Genderen & Pohl [9,10], image fusion '*is the combination of two or more different images to form a new image by using a certain algorithm' *e.g. integration of a large number of multivariate images from a remote sensing process into one image. Because Genderen & Pohl already stated PCA as a standard technique for image fusion in remote sensing, we adopt the more general definition of the term *image fusion *from the remote sensing community. Whereas in the medical imaging community the meaning of the term *image fusion *is commonly restricted to fusion of multimodal images, the definition of this term used in this article also includes multivariate images such as multispectral or multitemporal images.

Pattern recognition methods such as *artificial neural networks *(ANN) have gained much attention from the remote sensing community [11-15]. From the point of view of pattern recognition, the problem of image fusion is strongly related to the task of *dimension reduction*: Ignoring the spatial order of the patterns **x**, the image data is an unordered set of patterns that forms a data distribution in the data space  and image fusion or dimension reduction corresponds to a mapping



to a new low dimensional space  which retains certain properties of the original data distribution. Subsequently, the mapped patterns  can be spatially ordered according to the locations **p **of the corresponding sources, leading to the final fused images.

Well-known algorithms such as *Principal Component Analysis *(PCA) [16] or *Self Organising Maps *[17] have been successfully applied for various tasks of multispectral or multitemporal image fusion [11-15]. It is important to note that these methods are not bounded with limitations on the dimensionality of . Hence, they are especially suited if  is high dimensional.

In this work, we investigate the application of machine learning algorithms to medical image fusion. We compare the results of the standard linear PCA with it's nonlinear extension, the so called *Kernel PCA *(KPCA) which was proposed by Schölkopf et al. in 1998 [18]. Our empirical observations are presented and discussed by means of DCE-MRI data sets from a breast cancer screening study [19]. Image material presented in this paper is also provided online in original size (PNG format) [20].

## Methods

In the following, we briefly describe the theoretical background of the linear PCA and nonlinear KPCA algorithms and their application to the task of image fusion. Both methods determine a set of projection directions, referred to as *principal directions *(PDs), by optimising a certain criterion. The mapping *M *is defined by a subset of all possible PDs. Projecting each pattern **x**_**p **_on to one of these PDs associates each spatial position **p **with a new scalar value  (the *principal component*) of which integrates information from the different components  of **x**_**p**_, respectively. The resulting 3D image can be visualised as a gray value image or using perceptually optimised colour scales [21,22]. Alternatively, the low dimensional representation  of the patterns can be displayed as RGB composite images, if *M *is defined by a set of three PDs.

### Principal component analysis

Principal Component Analysis is one of the most frequently used dimension reduction method. Suppose the data are given by the set Γ = {**x**_*i*_}, **x**_*i *_∈ , 0 ≤ *i *≤ *N*, PCA is a transformation in a new coordinate system of uncorrelated and orthogonal principal axes ***ξ ***∈ , |***ξ***| = 1 which can be derived from the eigenvectors of the covariance matrix



by solving the eigenvalue equation

*λ****ξ ***= *C****ξ ***    (2)

for *λ *≥ 0 and ***ξ ***∈  \ {0}. The first eigenvector ***ξ***_1 _(the one with the largest eigenvalue *λ*_1_) maximises the variance . Therefore, the set of the first *n *≤ *d *eigenvectors or PDs carry more variance than any other *n *orthogonal projections.

### Kernel principal component analysis

In recent years, kernel based methods have been the object of much research effort within the machine learning community. The concept of a subset of kernel methods is based on the combination of well-known linear algorithms such as *Principal Component Analysis *or *Fisher Discriminant Analysis *with nonlinear kernel functions [23,24]. While the application of these functions allows more powerful nonlinear solutions, the kernelised algorithms retain most properties of their linear versions.

Consider a nonlinear function



which maps the examples **x **∈ Γ to some feature space [25]. Furthermore, assume that the mapped data are centred in . In order to perform the PCA in , one has to find the eigenvectors ***ξ ***of the covariance matrix



i.e. those vectors that satisfy  with ***ξ ***∈  \ {0} and *λ *≥ 0. Substituting (3), it is easy to see that the eigenvectors ***ξ ***lie in the span of Φ(**x**_1_),...,Φ(**x**_*N*_). Therefore, Schölkopf et al. [26] define the equivalent eigenvalue problem

*Nλ****α ***= *K****α***    (4)

where **α **denotes the column vector of coefficients *α*^(1)^,...,*α*^(*N*) ^describing the dual form of the eigenvector by



and *K *is the symmetric *Gram matrix *with elements

*K*_*ij *_= *K*(**x**_*i*_, **x**_*j*_) = Φ(**x**_*i*_), Φ(**x**_*j*_).    (6)

Normalising ***α***_*k *_corresponding to the k-th eigenvalue *λ*_*k *_of *K *ensures *λ*_*k*_***α***_*k*_, ***α***_*k*_ = 1. Now, principal components can be extracted in  by projecting an example **x **on ***ξ***_*k *_using



It is crucial to note that for extracting principal components using (4) and (7) the inner product Φ(**x**_*i*_), Φ(**x**_*j*_) is needed rather than the explicit images Φ(**x**_*i*_), Φ(**x**_*j*_) alone. Instead, one can use *kernel functions *fulfilling *Mercer's Theorem *such as the *Gaussian Kernel*



with bandwidth parameter *σ *or the *Polynomial Kernel of degree d*

*K*(**x**_*i*_, **x**_*j*_) = **x**_*i*_, **x**_*j*_^*d *^    (9)

which allow the PCA in the corresponding  to be performed implicitly with reasonable computational costs. For the Polynomial Kernel we have a clear interpretation of KPCA. In this case,  is the space of all monomials of degree *d *of the pattern components. Thus, KPCA is a linear PCA of the corresponding high order statistical features. The KPCA algorithm can be summarised as follows:

1. Calculate the Gram matrix *K *of Γ using a suitable parameterised kernel function.

2. Transform *K *according



with . This transformation implicitly moves the centre of mass of the mapped data {Φ(**x**_*i*_)}, **x**_*i *_∈ Γ to the origin of , i.e. centres the data in .

3. Calculate the eigenvector expansion coefficients ***α***_*k*_, i.e. the eigenvectors of  and normalise them.

4. Extract principal components using (7).

### Compression vs. discrimination

Application of both image fusion techniques leads to a set of up to *d *PDs in case of PCA and up to *N *PDs in case of KPCA. In general, a compact visualisation of the complete data as a single image is desired. In this case, inspection of the fused image based on the PD corresponding to the first (largest) eigenvalue is optimal in terms of a general compression scheme: The projection on this PD retains most of the total data variance and leads to a reconstruction with least mean square error. Nevertheless, image fusion is commonly employed with a well defined intention e.g. in order to detect a specific phenomenon such as bushfires in multitemporal satellite images [11] or (as in this work) tumour lesions in DCE-MRI data. In addition to the general compression characteristics, the fused image has to show task-specific discriminative properties which do not necessarily reflect the total data variance. In this case, using a PD corresponding to one of the following eigenvalues may lead to more discriminative visualisations. If the image data are fused by KPCA, an additional degree of freedom can be exploited. In addition to the index of the selected PD, the type and parameterisation of the kernel *K *can be varied leading to alternative mappings to the feature space, changing the characteristic of the fusion image.

### Experiments

In the following, the fusion results of both methods are discussed and illustrated with DCE-MRI sequences from six cases (referred to as *S*_1_,...,*S*_6_) which were taken during the the *MARIBS *breast screening study [19]. Each sequence consists of seven 3D MRI volumes of the female breast, recorded with a separation of 90 sec using a standardised protocol (A fast spoiled gradient echo sequence (FLASH) with TR = 12 ms, TE = 5 ms, ip angle = 35°, FOV = 340 mm and coronal slice orientation). Before recording the third volume, a gadolinium-based contrast agent was administered with a bolus injection. Therefore, each spatial position **p **in the 256 × 128 × 64 (1.33 mm × 1.33 mm × 2.5 mm) sized volume is associated with a pattern , *d *= 7 describing the temporal signal kinetic of the local tissue.

The images were manually evaluated by an expert who marked voxels of tumour with a cursor on an evaluation device. Below, the kinetic signals of the marked tumour voxels are labelled '+'. Signals corresponding to voxels of the complement of the marked region are labelled '-'.

For this kind of data, experiments of Lucht et al. [5] suggest recording a much longer temporal sequence of 28 images which makes the need for efficient fusion techniques evident.

#### Evaluation criteria

In order to provide an objective discussion of the value and drawbacks of both algorithms, we focus on the following requirements:

1. The marked region should be visualised with high contrast compared to unmarked regions in order to facilitate detection of kinetic signals which are similar to the marked signals.

2. The fusion image should follow the first criteria without time consuming manual manipulation by the observer (e.g. tuning of transfer functions such as windowing).

Following the first criteria, the purpose of the visualisation is specified implicitly by the voxel labels. In the present work, the expert marked regions of tumour tissues. Thus, optimal fusion images of an image sequence display locations of cancerous kinetic signals with high contrast to normal signals.

Next to the visualisation of the fusion images as gray value and RGB images, both methods are evaluated by means of a *receiver-operating-characteristic *(ROC) analysis [27,28]. To this end, pixel intensities of the fusion images are interpreted as confidence values for the existence of suspicious signals and are compared with the expert label as ground truth. The ROC analysis objectively measures the applicability of the fusion images for the task of lesion detection.

However, no conclusion can be drawn about how well other tissue types are distinguishable in the fusion images, i.e. how well the information of the entire signal space is represented.

#### Preprocessing

For numerical reasons, the voxel value range of each volume sequence is individually normalised to [0; 1]. In order to preserve the signal kinetics, the individual minimal and maximal intensity value is determined simultaneously on all *d *image volumes of each sequence. To ensure this normalisation is robust with respect to single outlier values, the values are calculated based on an application of a 3 × 3 × 3 median filter.

Since about 66% of each volume is covered by background, all images sequences are preprocessed with a full automatic tissue/background separation method. The histogram of the *sum of local intensity differences *(sod) feature



individually calculated for each sequence, has a bimodal shape and shows a clear separable maximum for the background voxels. The optimal threshold separating background from tissue can be computed automatically [29]. The resulting binary masks are postprocessed with a morphological *closing operator *[30] to ensure closed masks for the regions of tissue.

#### Adaptation

In order to automate and optimise the fusion process, a priori knowledge about the phenomenon to be visualised, given by the expert label, is used to find a suitable parameterisation of the algorithms as described in detail in the following section. In practice, these labels are not available for new image sequences. Thus, the algorithms have to be adapted on a small number of image sequences, e.g. from a subgroup of cases of a screening study, which were manually evaluated by a human expert and can be subsequently applied to the data of an arbitrary number of unseen cases.

To assure the experimental setup reflects the circumstances of a practical application, the data sets Γ used for adaptation consist of marked tissue signals from only five of the six image sequences and the sixth unseen image sequence is used for the evaluation of the algorithm's capabilities. This setup is repeated six times, each time using a different image sequence for evaluation. In case of KPCA, using all kinetic signals from the five image sequences is prohibitive due to the computational and memory complexity. Therefore, the KPCA is adapted with a reduced data set Γ consisting of all signals of the marked tumour regions and an equal number of signals randomly selected from non-tumour regions.

#### Parameter selection

An essential part of kernel methods is the mapping from the data space  to the feature space  by the kernel function. In this paper, we focus on the frequently used *Gaussian Kernel *(8) which is parameterised by the bandwidth parameter *σ*. Selection of this parameter is crucial for the fusion process. For the experiments, *s *is chosen by scanning the range [0.05,...,2.0] using a step size of 0.05. Because manual evaluation by visual examination of the fusion images of each parameterisation is time consuming, we apply an automatic selection heuristic for the bandwidth based on the component specific *Fisher score*



with class specific mean *μ*^± ^and variance *v*^±^. The Fisher score is commonly used for ranking components *x*^(*k*) ^of a set {(**x**, *y*)} of binary labelled (*y *= ±) examples according to their discriminative power. In a similar manner, the score can be evaluated for different PDs on a random subset of the training set Γ utilising the corresponding principal component values with their associated expert label and thus can be interpreted as a measure for the first evaluation criteria. Furthermore, the sign of the PCA/KPCA based PDs can be adjusted in order to obtain a high value for the average intensity of tumour voxels causing tumour lesions to appear as bright regions.

Thereby, the a priori knowledge of which region of the five image sequences used for adaptation should be visualised with high contrast can be utilised for selecting proper parameterisations which lead to discriminative visualisations tailored to the given task.

### Fusion

**Figure 10:**
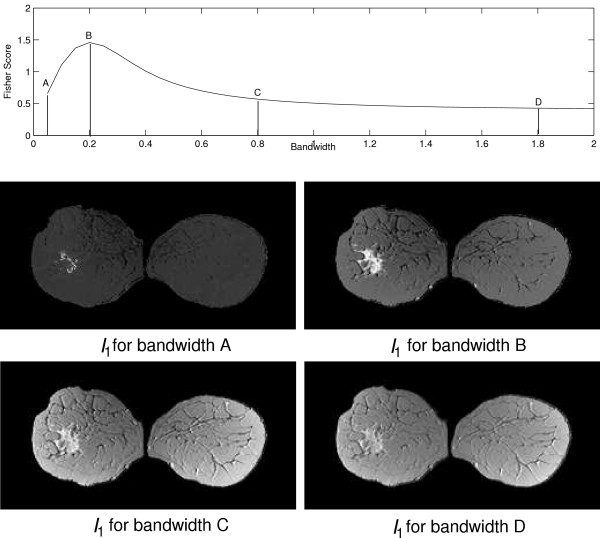
Plot of Fisher score values for PD_1 _of the KPCA algorithm with varying bandwidth. The score indicates a varying magnitude of separation between the class of suspicious tissue signals and the class of normal tissue signals. Below, the fusion image *I*_1 _for *S*_1 _based KPCA with four different bandwidth values A, B, C and D is shown. Variation of the bandwidth leads to fusion images with varying imaging properties. The bandwidth B leads to a fusion image that displays the tumour with the highest contrast to the surrounding tissue and the Fisher score shows a peak at the corresponding position. For bandwidth values A, C and D, the Fisher score and the contrast in the fusion images decreases.

For each method and image sequence, the first three PDs are used for calculating fused images, referred to as *I*_1_, *I*_2 _and *I*_3_. For the purpose of visualisation, the range of the voxel values is normalised to [0; 255]. Additionally, *I*_1_, *I*_2 _and *I*_3 _are composed in to an RGB image *I*_*RGB*_. For fusion images based on KPCA, the bandwidth for each *I*_*k *_is chosen according to the individual maximum of the Fisher criterion as illustrated in Fig. 10.

## Results

**Figure 4:**
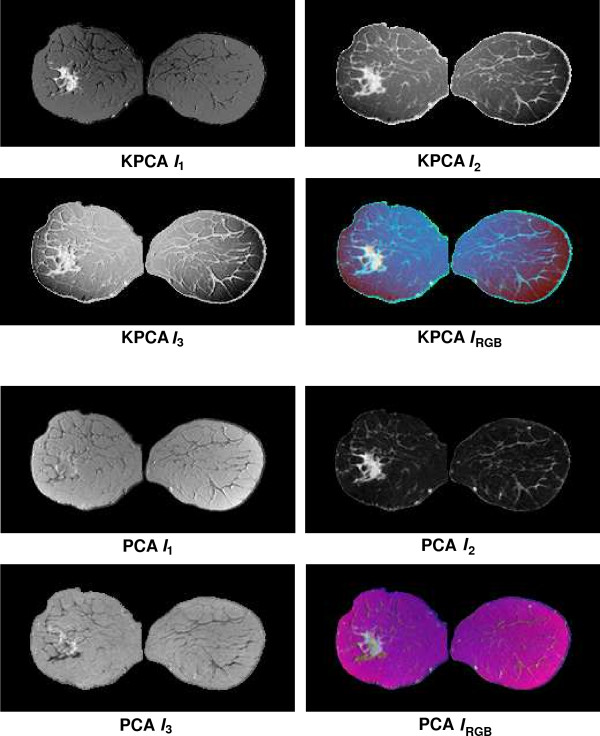
Fusion images *I*_1_, *I*_2_, *I*_3 _and corresponding colour composite image *I*_*RGB *_for sequence *S*_1 _based on KPCA (upper 2 × 2 block) and PCA (lower 2 × 2 block). The lesion is located near the centre of the left breast.

**Figure 5:**
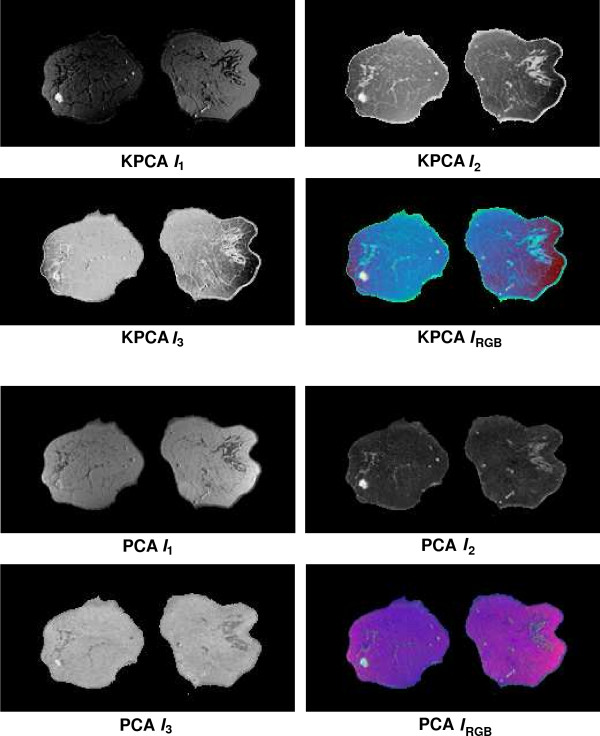
Fusion images *I*_1_, *I*_2_, *I*_3 _and corresponding colour composite image *I*_*RGB *_for sequence *S*_2 _based on KPCA (upper 2 × 2 block) and PCA (lower 2 × 2 block). The lesion is located in the lower left part of the left breast.

**Figure 6:**
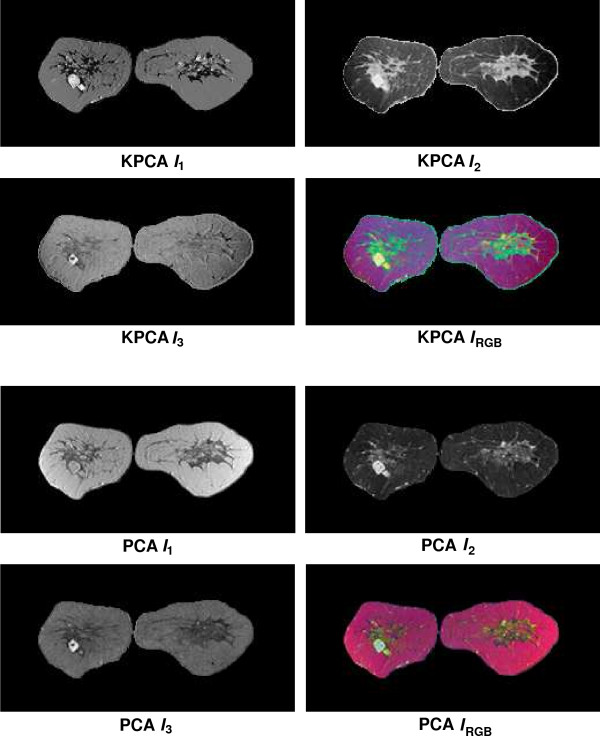
Fusion images *I*_1_, *I*_2_, *I*_3 _and corresponding colour composite image *I*_*RGB *_for sequence *S*_3 _based on KPCA (upper 2 × 2 block) and PCA (lower 2 × 2 block). The lesion is located near the centre of the left breast.

**Figure 7:**
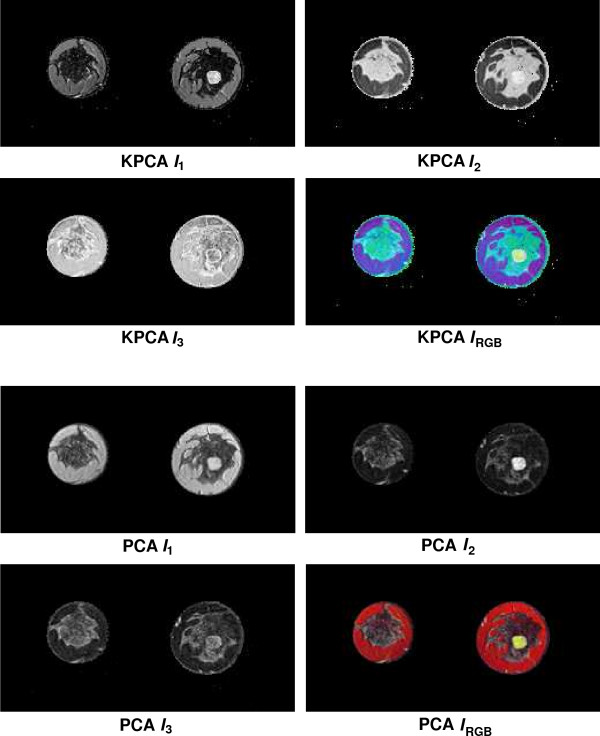
Fusion images *I*_1_, *I*_2_, *I*_3 _and corresponding colour composite image *I*_*RGB *_for sequence *S*_4 _based on KPCA (upper 2 × 2 block) and PCA (lower 2 × 2 block). The lesion is located near the centre of the right breast.

**Figure 8:**
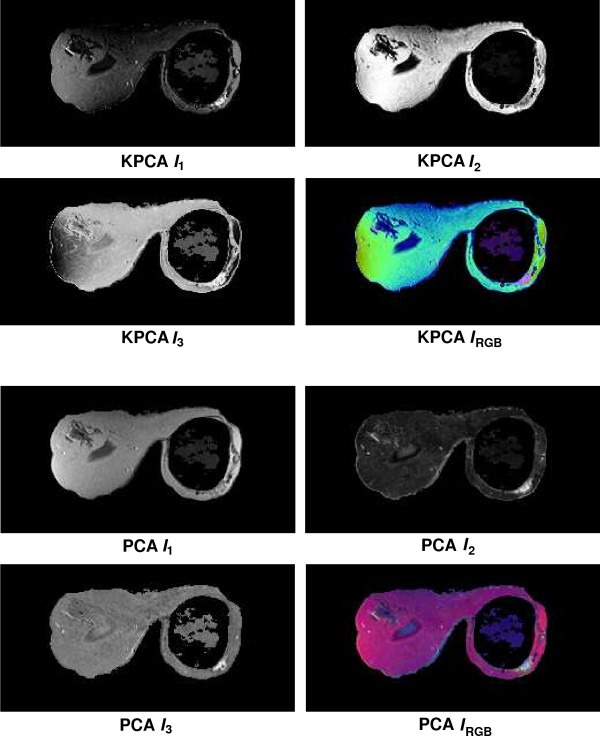
Fusion images *I*_1_, *I*_2_, *I*_3 _and corresponding colour composite image *I*_*RGB *_for sequence *S*_5 _based on KPCA (upper 2 × 2 block) and PCA (lower 2 × 2 block). The lesion is located near the implant in the right breast.

**Figure 9:**
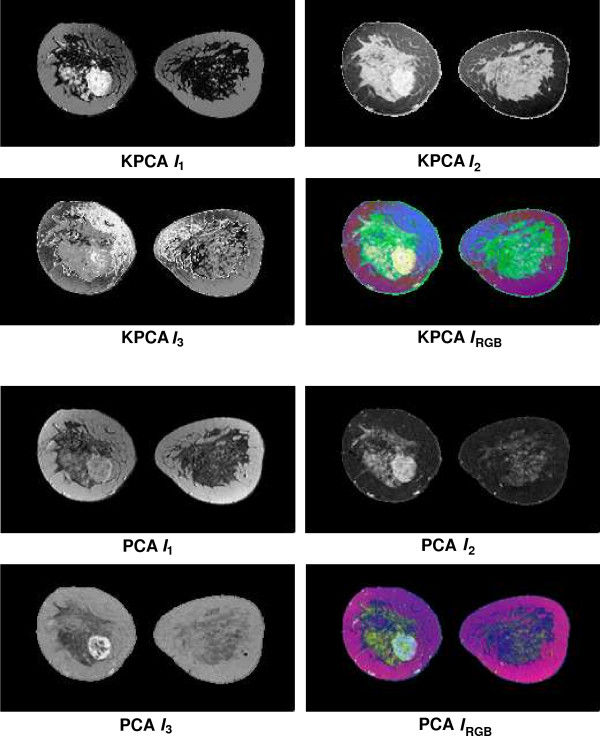
Fusion images *I*_1_, *I*_2_, *I*_3 _and corresponding colour composite image *I*_*RGB *_for sequence *S*_6 _based on KPCA (upper 2 × 2 block) and PCA (lower 2 × 2 block). The lesion is located near the centre of the left breast and is surrounded by glandular tissue.

Fusion results for the sequences *S*_1_,...,*S*_6 _based on the PCA algorithm are shown in the lower 2 × 2 block of Fig. 4, Fig. 5, Fig. 6, Fig. 7, Fig. 8 and Fig. 9. For all six sequences, the fusion image *I*_1 _based on the PD with the leading eigenvalue does not lead to discriminative visualisations. The tumour lesions appear with the same intensity as fatty tissue, while glandula tissue is displayed as dark areas (*S*_3_, *S*_4_). In contrast to *I*_1_, the discriminative power of *I*_2 _is obviously much greater for all six image sequences. The display of the tumour lesions (high intensity values) differs significantly from areas of glandular tissues, blood vessels (medium intensity values) and fatty tissue (low intensity values). The contrast between tumour lesion and the surrounding tissue decreases in *I*_3 _of *S*_2_, *S*_3 _and *S*_5_. Additionally, the surrounding tissue is displayed less detailed (*S*_1_, *S*_2_, *S*_4_, *S*_5_). According to the weak discriminative characteristic of *I*_1 _and *I*_3_, the tumour lesions are coloured with shadings of green or cyan in the corresponding *I*_*RGB*_.

Fusion images based on KPCA are shown in the upper 2 × 2 block of Fig. 4, Fig. 5, Fig. 6, Fig. 7, Fig. 8 and Fig. 9. For *S*_1_, *S*_2_, *S*_3 _and *S*_4_, image *I*_1 _displays the tumour lesion with high contrast to the surrounding tissues. Adipose tissue appears in *I*_1 _and *I*_2 _with mediumin tensity. In *I*_2 _of *S*_4 _and *S*_3_, glandular tissue can be observed in addition to the tumour. These areas appear dark in *I*_1_. The fraction of glandular tissue regions in *I*_2 _of *S*_1 _and *S*_2 _is much smaller, since the tumour is located near the chest muscle where the breast mostly consists of fatty tissue and blood vessels.

An interesting detail can be observed in *I*_3 _of *S*_4_. The image clearly shows a ring structure as part of or around the tumour lesion. At positions inside the ring which are displayed with high intensity values in *I*_1 _and *I*_2_, the temporal kinetic patterns show a fast uptake with a following constant or slightly decreasing concentration of the contrast agent. In contrast to the signals inside the ring, all signals corresponding to the ring structure in *I*_3 _show as teadily increasing concentration. In all composite images *I*_*RGB *_except for *S*_5_, the tumour lesions are coloured white and can be easily discriminated from fatty tissue (shadings of blue to purple) and glandular tissue (shadings of blue to green). For image *S*_5_, only *I*_1 _shows a discriminative characteristic. The tumour is displayed as a small cluster of high intensity values in the lower right area of the right breast, next to the implant.

According to common practice, the curves obtained from the ROC analysis of the fusion images *I*_1_, *I*_2 _and *I*_3 _are compared by measuring the *area*-*under*-*the*-*curve*(AUC) values. The corresponding AUC values are listed in Tab. 1. The fusion image yielding the highest AUC value is printed bold for each sequence. For five of six sequences, a fusion image based on PCA yields the highest AUC value (column *PCA *in Tab. 1). The fusion image *I*_2 _based on the second PD of the PCA algorithm significantly outperforms the corresponding PCA based fusion images *I*_1 _and *I*_3_. A similar predominance of *I*_2 _can be observed for the KPCA based AUC values (column *KPCA *in Tab. 1). Here, *I*_2 _outperforms *I*_1 _and *I*_3 _in four of six cases (*S*_1_, *S*_2_, *S*_3 _and *S*_5_). Only for *S*_4 _and *S*_6 _the fusion image *I*_1 _yields the largest AUC value. Nevertheless for KPCA, the difference to the corresponding fusion images *I*_1 _and *I*_3 _is much less distinct. In particular *I*_1 _yields AUC values which are close to those of the corresponding fusion image *I*_2_. The predominance of the second component also decreases, if the PCA algorithm is trained with the reduced data set used for adaptation of the KPCA (column *PCA (reduced) *in Tab. 1). In comparison with the results of the PCA adapted with the entire data set, the AUC values of *I*_2 _decrease and increase for the fusion images *I*_1 _and *I*_3_.

**Table 1:** Area under ROC curve values for fusion images *I*_1_, *I*_2 _and *I*_3 _for series *S*_1_,...,*S*_6 _based on KPCA, PCA and PCA trained with the same reduced training set as KPCA. For each AUC value, the pixel intensities of the fusion images are interpreted as confidence values indicating the existence of suspicious signals at the corresponding positions. The largest AUC value for each case is printed bold.

	**Area-Under-ROC-Curve**								
	KPCA			PCA			PCA (reduced set)		

Sequence	*I*_1_	*I*_2_	*I*_3_	*I*_1_	*I*_2_	*I*_3_	*I*_1_	*I*_2_	*I*_3_

*S*_1_	0.950	0.972	0.879	0.539	**0.993**	0.633	0.772	0.972	0.692
*S*_2_	0.918	0.945	0.728	0.727	**0.993**	0.712	0.852	0.948	0.547
*S*_3_	0.995	**0.998**	0.710	0.520	0.997	0.926	0.799	0.997	0.747
*S*_4_	0.996	0.985	0.259	0.926	**0.999**	0.919	0.992	0.985	0.963
*S*_5_	0.959	0.966	0.904	0.693	**0.997**	0.925	0.814	0.964	0.344
*S*_6_	0.994	0.986	0.706	0.926	**0.999**	0.919	0.785	0.986	0.802

The influence of the bandwidth *σ *on the fusion characteristic is illustrated in Fig. 10. For small values of the bandwidth *σ *only a small fraction of the tumour lesion appears with high intensities. If the bandwidth is chosen according to the maximum of the Fisher score, the lesion is visualised with high contrast to the surrounding tissue. In the shown example, the Fisher criterion decreases along with the contrast of the visualisation for further increasing bandwidth values.

## Discussion

The results shown in the preceding section indicate that fusion of DCE-MRI data by PCA or KPCA leads to compact and meaningful visualisations. Lesions are correctly displayed as bright regions or with specific colouring and can be easily discriminated from surrounding tissue. Once a small subgroup of cases is evaluated, the obtained secondary information in the form of labelled tumour areas is utilised for automation of the data processing and presentation: (*i*) The sign of the PD is selected in a way that tumour lesions always appear with high intensities. (*ii*) The parametrisation of the kernel function of the KPCA is optimised in such a way that the fusion images show the desired discriminative characteristics. Thus, both evaluation criteria stated in the section *Evaluation criteria *are accomplished.

**Figure 13:**
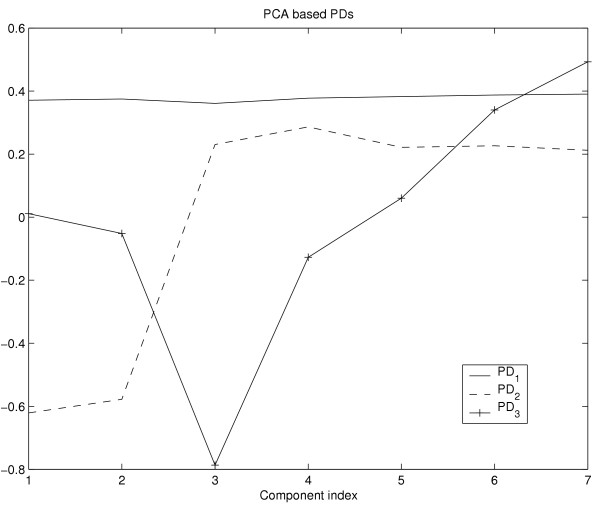
Plot of the components of the vectors PD_1 _(solid), PD_2 _(dashed) and *PD*_3 _(solid with crosses) based on the PCA algorithm. The plot of *PD*_2 _shows a typical signal of suspicious tissue (see Fig. 3) and therefore leads to discriminative fusion images with high intensity values at positions of tissue that exhibits a significant signal uptake after injection of the contrast agent.

Although both methods are applicable for the task of image fusion, several properties should be discussed in more detail. According to the ROC analysis and visual appraisal, the fusion image *I*_2 _based on PCA shows for nearly all cases a discriminative characteristic which is superior to all other fusion images based on PCA or KPCA. While *I*_1 _based on PCA captures the slightly increasing elucidation of the major part of the breast, caused by minor accumulation of contrast agent in tissues such as fat, the fusion image *I*_2 _corresponding to the second PD of PCA shows the lesions with high contrast to the surrounding tissue. This can also be observed by means of the PDs itself. Figure 13 shows a plot of the components of the three PCA based PDs. The plot of PD_1 _shows anearly constant or slightly increasing curve, whereas the plot of the components of PD_2 _is similar to a typical temporal course of contrast agent concentration insuspicious tissue (see Fig. 3). The plot of PD_3 _shows increasing values for the components corresponding to the postcontrast measurements. From this follows that the major part of the signal variance is caused by voxels which exhibit signals at different intensity levels with only minor changes of intensity in the course of time. This fraction of data variance is captured by PD_1 _of PCA. The next major source of variance is the signal uptake between the precontrast and the first postcontrast measurement insuspicious tissue which is captured by PD_2 _and leads to the superior discriminative characteristics of the fusion image *I*_2_. PD_3 _is sensitive to signals which show a continuously increasing intensity for the postcontrast measurements. Hence, *I*_3 _is more discriminative than *I*_1_, but less discriminative than *I*_2_.

**Figure 11:**
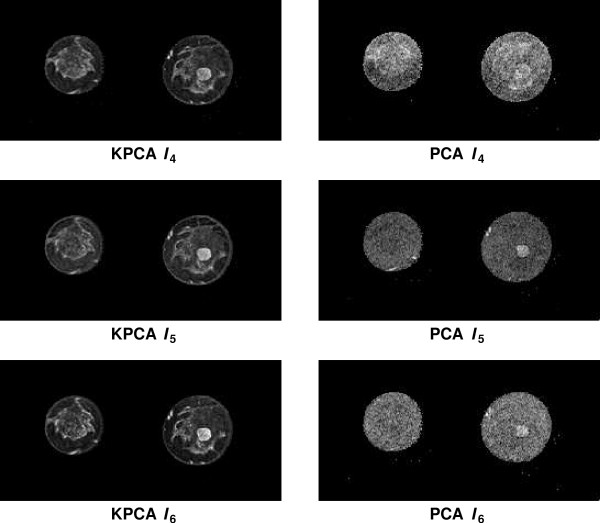
Fusion images *I*_4_, *I*_5 _and *I*_6 _for *S*_3 _based on the PDs with the fourth, fifth and sixth largest eigenvalue. The left column shows the fusion images based on KPCA. Each fusion image was calculated with a bandwidth that was individually optimised according to the Fisher score. The right column shows the same images fused with PCA. In contrast to the KPCA based fusion images, these images show a significant fraction of high frequent noise and less details.

The ROC analysis of the KPCA based fusion images indicates that the fusion images *I*_2 _show superior discriminative characteristics for four of six cases (*S*_1_, *S*_2_, *S*_3 _and *S*_5_). However, selection of a suitable kernel parametrisation leads to comparable AUC values for *I*_1_. For fusion images corresponding to PDs with smaller eigenvalues, KPCA based images still show more details than those based on PCA, if the bandwidth value is chosen according to the maximum of the Fisher score. Figure 11 shows the KPCA based (left column) and the PCA based (right column) fusion images *I*_4_, *I*_5 _and *I*_6 _for sequence *S*_4_. While KPCA distributes the total data variance on *N *PDs, the PCA method uses only *d *PDs. Therefore, the PCA based fusion images *I*_4_, *I*_5 _and *I*_6 _typically contain a large fraction of high frequent noise. It is important to note that the fusion images based on KPCA are not necessarily uncorrelated, if each image is calculated using PDs with different bandwidth values, and therefore may display redundant information. In five of six cases, RGB visualisations based on KPCA show the tumour lesion as white regions which are easy to discriminate from other tissue types. In contrast to subtraction images which also allow detection of lesions with high sensitivity (see e.g. [4]), the fusion images *I*_*RGB *_provide a more comprehensive display of the data. A single subtraction image displays only the information of a two dimensional subspace of the signal space , i.e. the information of two manually selected components of the signal vector. Without further manipulation of the transfer function and after selection of two suitable components, a subtraction image commonly shows the lesion as a cluster of high intensity values and other types of tissue are not displayed or indistinguishable. The fusion images are low dimensional representations of the entire signal space. Thus, the RGB composite images *I*_*RGB *_based on PCA or KPCA clearly display the lesion in combination with glandular or fatty tissue and major blood vessels.

**Figure 12:**
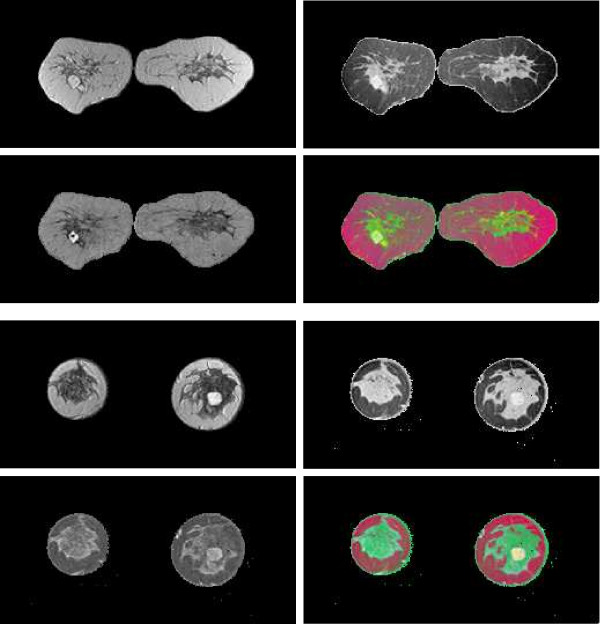
Image *I*_1_, *I*_2_, *I*_3 _and *I*_*RGB *_of *S*_3_(top block) and *S*_4_(bottom block) fused by the PCA algorithm which was adapted on the same reduced data set as KPCA.

**Figure 14:**
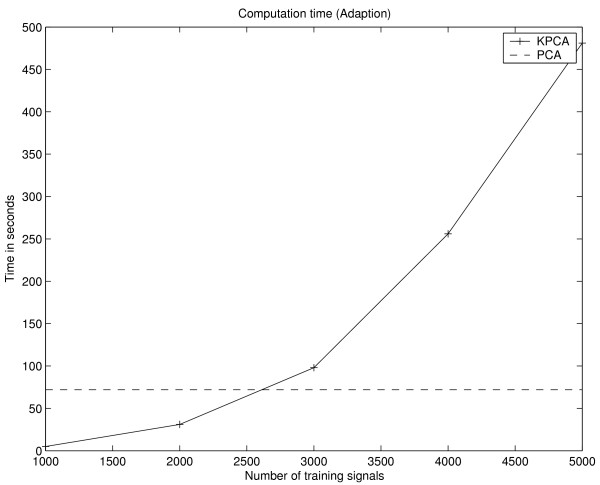
Computation time for adaptation of KPCA (solid line). The measured time includes calculation of the kernel matrix and the extraction of the first three PDs. Additionally, the time for adaptation of the PCA using the complete training data is shown (dashed line).

One drawback of KPCA is the increased computational and memory complexity in contrast to PCA. In case of KPCA, the complexity scales with the size *N *of the training set Γ. During the adaptation of KPCA, an *N *× *N *sized kernel matrix has to be stored and manipulated, whereas the covariance matrix for PCA is only of size *d *× *d*. Thus for KPCA, the computation time (LINUX system / 1.8 GHz Pentium IV / 2 GB RAM) for the adaptation, i.e. calculation of the kernel matrix and extraction of 3 PDs, increases significantly with the size of the training set Γ and takes 73 seconds for Γ consisting of 2700 training items which is comparable to the computation time of the PCA for the given setup (see Fig. 14). While even for large matrices, a subset of eigenvectors can be extracted in a reasonable time using efficient numerical software packages like LAPACK [31], the memory complexity obviously limits the size of Γ. One way to address this problem is to subsample the data. Instead of using a random sample of the whole data set, the chosen scheme assures the presence of tumour voxels in the training set. In the former case, the presence of a larger number tumour voxels is unlikely because of the unbalanced ratio between number of tumour voxels and the number of non-tumour voxels. Nevertheless, the reduction of the training data causes a degradation of the detection performance and changing fusion characteristics (see Fig. 12).

**Figure 15:**
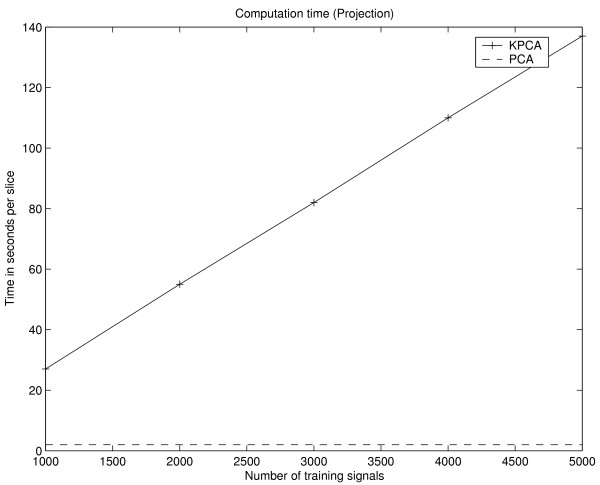
Computation time for the three fusion images *I*_1_, *I*_2 _and *I*_3 _of one slice using PCA (dashed line) and KPCA (solid line). The computation time of principal component values with KPCA increases linearly with the size of the training set. For PCA, the computation time depends only on the dimension of the signal pattern and is constant for the given setup.

More important for practical applications of both methods is the computational expense for calculation of the fusion images. Using PCA, the value of a fusion image voxel is equivalent to the inner product of two *d*-dimensional vectors and the calculation of the three fusion images *I*_1_, *I*_2 _and *I*_3 _of one volume slice takes approximately 1 second. In case of KPCA, the inner product has to be calculated in the feature space and the PD in  is only implicitly given as an expansion of *N *kernel functions. Thus, computation of *I*_1_, *I*_2 _and *I*_3 _of one volume slice takes approximately 23 seconds for training sets Γ consisting of 1000 examples and increases linearly with the size of Γ (Fig. 15).

In consideration of the fact that both methods are able to fuse the multitemporal DCE-MRI to single meaningful images which do not only show the lesion with high intensities, but also other types of tissue such as fatty or glandular tissue, the standard linear PCA seems to be most suitable for the given signal domain because of it's low computation time and superior detection performance. Only for PCA, the three fusion images can be calculated for a complete volume in a reasonable time and without delaying the diagnostic process. According to the ROC analysis, the introduction of nonlinearity by the kernel function did not improve the discriminative properties of the fusion images, but visual appraisal of the RGB composite images based on KPCA suggest a more comprehensive display of the different types of tissue. It is an open question whether fusion images of other data domains with more complex or higher dimensional signals might benefit more obviously from the nonlinearity of KPCA.

## Conclusion

In this paper, we have demonstrated the integration of distributed information from DCE-MRI image sequences to meaningful visualisations by means of PCA and KPCA. Both methods were able to accentuate the regions marked by the expert as important in image sequences blinded to automatic analyses. By the employment of task-specific information, the parametrisation of the KPCA algorithm was optimised in order to accentuate the relevant characteristics of the visualisation.

## List of abbreviations

**PCA **Principal Component Analysis

**KPCA **Kernel Principal Component Analysis

**PD **Principal Direction

**DCE-MRI **Dynamic Contrast Enhanced Magnetic Resonance Imaging

## Authors' contributions

T. Twellmann, A. Saalbach and T. W. Nattkemper conceived the experimental setup. Implementation and realisation was done by O. Gerstung. Image acquisition was done under supervision of M. O. Leach.
